# DISCORDANCE IN GOALS OF CARE BETWEEN PATIENTS WITH ADVANCED CANCER AND THEIR CAREGIVERS: DOES AGE PLAY A ROLE?

**DOI:** 10.1093/geroni/igz038.211

**Published:** 2019-11-08

**Authors:** Amy R Lipson, Sara Douglas

**Affiliations:** Case Western Reserve University, Cleveland, Ohio, United States

## Abstract

Cancer is considered a family disease as the caregivers (CG’s) role extends beyond providing care as they can also help facilitate treatment decisions. While much has been reported in the literature about patient (PT) goals of care (GoC), little is known about discordance between PT and CG GoC and the impact of PT age. The variables of interest were PT and CG identified GoC using a 100-point visual analog scale (VAS) with anchors of quality of life (0) and survival (100). Discordance was defined as a > 40 point difference on the VAS. The GoC data reported here were those obtained at enrollment and prior to subject’s death. A sample of 235 PTs and CGs of PTs diagnosed with advanced cancers were included in the study. Mean age for the PTs was 64.7 (SD=10.5, range =21-88) with 54% being > 65. At enrollment, 28.7% of the PT-CG pairs of those PTs 65 years (X2 (1)=1.06, p=.304). At death, 61.8% (X2 (1)=31.04 <.001, Φ=.49) with discord at enrollment had discord at death. For patients who were older, 66.7% who had discord at enrollment also had discord at death and for patients

